# Supplementary material for: Nonlinear amplitude dynamics in flagellar beating
Source: R Soc Open Sci. 2017 Mar 8;4(3):160698. doi: 10.1098/rsos.160698 (PMC5383814; doi:10.1098/rsos.160698)
Supplement: Supplementary Text [file rsos160698supp1.pdf]

Nonlinear amplitude dynamics in flagellar beating (Supplementary Text)

David Oriola,[1,*] Hermes Gadêlha,[2,3] Jaume Casademunt [1]

[1]Departament de Física de la Matèria Condensada
Facultat de Física, Universitat de Barcelona
Avinguda Diagonal 647, E-08028 Barcelona, Spain.
[2]Department of Mathematics, University of York, YO10 5DD, UK.
[3]Wolfson Centre for Mathematical Biology, Mathematical Institute
University of Oxford, Oxford OX2 6GG, UK.

*Current address:
Max Planck Institute of Molecular Cell Biology and Genetics
Pfotenhauerstraße 108, 01307
&
Max Planck Institute for the Physics of Complex Systems
Nöthnitzerstraße 38, 01187
Dresden, Germany

To whom correspondence should be addressed; E-mail: oriola@mpi-cbg.de, oriola@pks.mpg.de.

1 Nonlinear elastohydrodynamic flagella equations

Here we provide the derivation of the nonlinear elastohydrodynamic flagella equations by using a formalism based on the special theory of Cosserat rods (*1*). An alternative derivation can be found in Ref. (*2*) by using the minimization of an energy functional for the flagellum. Finally we discuss how boundary conditions are obtained considering the case of clamped condition at the base.

The equilibrium equations for a rod subject to general contact forces $\boldsymbol{N}(s,t)$ and contact moments $\boldsymbol{M}(s,t)$ reads (*1*):

$$\boldsymbol{N}_s + \boldsymbol{F}_{\text{ext}} = \boldsymbol{0} \tag{1}$$

$$\boldsymbol{M}_s + \hat{\boldsymbol{s}} \times \boldsymbol{N} + \boldsymbol{L}_{\text{ext}} = \boldsymbol{0} \tag{2}$$

where $\boldsymbol{F}_{\text{ext}}$, $\boldsymbol{L}_{\text{ext}}$ are general external forces and torques. The internal moment of the bundle $\boldsymbol{M}(s,t)$ reads:

$$\boldsymbol{M} = (E_b\phi_s - bF)\hat{\boldsymbol{k}} \tag{3}$$

where $F(s,t) = \int_s^L f(s',t)ds'$. Differentiating the last expression respect to the arc length we have:

$$\boldsymbol{M}_s = \hat{\boldsymbol{s}} \times [(E_b\phi_{ss} + bf)\hat{\boldsymbol{n}} + \tau\hat{\boldsymbol{s}}] \tag{4}$$

where $\tau(s,t)$ is the tension acting along the flagellum. In the absence of external torques ($\boldsymbol{L}_{\text{ext}} = 0$) and using Eq. 2 we obtain the resultant contact force:

$$\boldsymbol{N} = -(E_b\phi_{ss} + bf)\hat{\boldsymbol{n}} + \tau\hat{\boldsymbol{s}} \tag{5}$$

Differentiating the contact force respect to the arc length we have:

$$\boldsymbol{N}_s = (-E_b\phi_{sss} - bf_s + \phi_s\tau)\hat{\boldsymbol{n}} + (E_b\phi_s\phi_{ss} + b\phi_s f + \tau_s)\hat{\boldsymbol{s}} \tag{6}$$

The flagellum is immersed in a fluid where we will consider the low Reynolds number approximation. The viscous drag force $\boldsymbol{F}_{\text{vis}}$ is given by resistive force theory:

$$\boldsymbol{F}_{\text{vis}} = -\zeta_{\perp}(\hat{\boldsymbol{n}} \cdot \boldsymbol{r}_t)\hat{\boldsymbol{n}} - \zeta_{\parallel}(\hat{\boldsymbol{s}} \cdot \boldsymbol{r}_t)\hat{\boldsymbol{s}} \tag{7}$$

Using Eq. 1 and considering $\boldsymbol{F}_{\text{ext}} = \boldsymbol{F}_{\text{vis}}$ we have:

$$\boldsymbol{r}_t \;=\; \frac{1}{\zeta_{\parallel}}(E_b\phi_s\phi_{ss} + b\phi_s f + \tau_s)\hat{\boldsymbol{s}} + \frac{1}{\zeta_{\perp}}(-E_b\phi_{sss} - bf_s + \phi_s\tau)\hat{\boldsymbol{n}} \tag{8}$$

Using the fact that $\hat{\boldsymbol{s}}_t = \phi_t\,\hat{\boldsymbol{n}}$ we obtain an equation for ϕ:

$$\phi_t \;=\; \frac{1}{\zeta_{\parallel}}\phi_s(E_b\phi_s\phi_{ss} + b\phi_s f + \tau_s) + \frac{1}{\zeta_{\perp}}(-E_b\phi_{ssss} - bf_{ss} + \phi_{ss}\tau + \phi_s\tau_s) \tag{9}$$

An equation for the tension can be obtained by using the inextensibility condition $\partial_t(\hat{\boldsymbol{s}} \cdot \hat{\boldsymbol{s}}) = 2\hat{\boldsymbol{s}}_t \cdot \hat{\boldsymbol{s}} = 0$. The differential equation for the tension reads:

$$\tau_{ss} - \frac{\zeta_{\parallel}}{\zeta_{\perp}}(\phi_s)^2\tau + E_b\partial_s(\phi_s\phi_{ss}) + b\partial_s(\phi_s f) + \frac{\zeta_{\parallel}}{\zeta_{\perp}}\phi_s(E_b\phi_{sss} + bf_s) = 0 \tag{10}$$

Next we non-dimensionalize the last equations as described in the Main Text. Additionally, we define $\bar{\zeta} \equiv \zeta_{\perp}/\zeta_{\parallel}$ and we non-dimensionalize the tension with respect to E_b/L^2. Finally, the dimensionless equations read:

$$\mathrm{Sp}^4\phi_t \;=\; \bar{\zeta}[(\phi_s)^2(\phi_{ss} + \mu_a f) + \tau_s\phi_s] - \phi_{ssss} - \mu_a f_{ss} + \phi_{ss}\tau + \phi_s\tau_s \tag{11}$$

$$\tau_{ss} - \frac{1}{\bar{\zeta}}(\phi_s)^2\tau = -\partial_s(\phi_s\phi_{ss}) - \mu_a\partial_s(\phi_s f) - \frac{\phi_s}{\bar{\zeta}}(\phi_{sss} + \mu_a f_s) \tag{12}$$

1.1 Boundary conditions

We need to specify the contact moment and the contact force at the boundaries. At $s = 0$ we have:

$$\begin{aligned} \boldsymbol{M}_{\text{ext}}|_{s=0} &= [-E_b\phi_s|_{s=0} + bF(0,t)]\hat{\boldsymbol{k}} \\ \boldsymbol{N}_{\text{ext}}|_{s=0} &= [E_b\phi_{ss}|_{s=0} + bf(0,t)]\hat{\boldsymbol{n}} - \tau(0,t)\hat{\boldsymbol{s}} \end{aligned} \tag{13}$$

At $s = L$ we have:

$$\begin{aligned} \boldsymbol{M}_{\text{ext}}|_{s=L} &= E_b\phi_s|_{s=L}\hat{\boldsymbol{k}} \\ \boldsymbol{N}_{\text{ext}}|_{s=L} &= -[E_b\phi_{ss}|_{s=L} + bf(L,t)]\hat{\boldsymbol{n}} + \tau(L,t)\hat{\boldsymbol{s}} \end{aligned} \tag{14}$$

Next we switch to dimensionless variables where the external contact moment is scaled by E_b/L, and external contact force and tension by E_b/L^2. At $s = 0$ we have:

$$\begin{aligned} \boldsymbol{M}_{\text{ext}}|_{s=0} &= [-\phi_s|_{s=0} + \mu_a F(0,t)]\hat{\boldsymbol{k}} \\ \boldsymbol{N}_{\text{ext}}|_{s=0} &= [\phi_{ss}|_{s=0} + \mu_a f(0,t)]\hat{\boldsymbol{n}} - \tau(0,t)\hat{\boldsymbol{s}} \end{aligned} \tag{15}$$

where now $F(s,t) = \int_s^1 f(s',t)ds'$. At $s = 1$ we have:

$$\begin{aligned} \boldsymbol{M}_{\text{ext}}|_{s=1} &= \phi_s|_{s=1}\hat{\boldsymbol{k}} \\ \boldsymbol{N}_{\text{ext}}|_{s=1} &= -[\phi_{ss}|_{s=1} + \mu_a f(1,t)]\hat{\boldsymbol{n}} + \tau(1,t)\hat{\boldsymbol{s}} \end{aligned} \tag{16}$$

We now consider the case of small curvature where $\phi_s \ll 1$ and $\tau \approx 0$. At the distant boundary condition we have no applied contact force or contact moment thus $\boldsymbol{M}_{\text{ext}}|_{s=1} = \boldsymbol{N}_{\text{ext}}|_{s=1} = \boldsymbol{0}$. This lead to the conditions $\phi_s|_{s=1} = 0$ and $\phi_{ss}|_{s=1} = -\mu_a f(1,t)$ respectively. The external contact force and contact moment at the base $\boldsymbol{N}_{\text{ext}}|_{s=0} = \boldsymbol{F}_{\text{head}}$, $\boldsymbol{M}_{\text{ext}}|_{s=0} = \boldsymbol{M}_{\text{head}}$ are given by the specific viscous fluid dynamics assumed. By considering a clamped condition, the base is fixed $\boldsymbol{r}_t|_{s=0} = 0$, and we obtain the condition $\phi_{sss}|_{s=0} = -\mu_a f_s|_{s=0}$. Additionally, the base is clamped and thus $\boldsymbol{M}_{\text{ext}}|_{s=0} = 0$. In the linear analysis, the four boundary conditions in Fourier space read:

$$\begin{aligned} \tilde{\phi}(0) &= 0 \\ \tilde{\phi}_{sss}(0) &= -\bar{\chi}\tilde{\phi}_s(0) \\ \tilde{\phi}_s(1) &= 0 \\ \tilde{\phi}_{ss}(1) &= -\bar{\chi}\tilde{\phi}(1) \end{aligned} \tag{17}$$

Concerning the constraints on the dynein distribution due to the boundary conditions, at $s = 0$ we have that $\phi_t|_{s=0} = 0$ and thus both plus and minus distributions decay exponentially with characteristic time $\bar{\tau}$ to n_0 at the steady state. For the case of $s = 1$, $\phi_t|_{s=1}$ is different from zero in general, thus the specific boundary conditions for ϕ at the tail constraint the evolution of dynein bound motor distributions.

2 Numerical integration of the nonlinear flagella equations

Here, we provide the numerical algorithm to numerically solve the nonlinear Eqs. 2.10 and 2.11 in the Main Text. We consider a uniform discretization in the arc length s of the bundle centerline with M intervals of step size $\Delta s = 1/M$. The discrete points are denoted $s_m = (m-1)\Delta s, m = 1, \ldots, M+1$ and the time is discretized as $t_n = n\Delta t$. Any continuous function $X(s,t)$ is denoted X_m^n in the discretized version. The study is done with $\Delta s = 2.5 \cdot 10^{-4}$ and $\Delta t = 5 \cdot 10^{-5}$ (dimensionless units).

2.1 Tangent angle dynamics

We will use a first-order IMEX (implicit-explicit) scheme for the integration of the tangent angle ϕ in the very first time step ($n = 0$) and a second-order IMEX scheme for $n \geq 1$ (*3*). After discretization, the problem reduces to a linear system of equations of the form $\mathrm{A}\phi^{n+1} = b$, where A is a $(M+1) \times (M+1)$ matrix and b, ϕ^{n+1} are $M+1$ vectors.

In the first time step ($n = 0$) we will use a first order IMEX scheme. The elements of the matrix A corresponding to the rows $m = 3, \ldots, M-1$ take the form:

$$[\mathrm{A}]_{mm'} = \delta_{mm'} + \alpha[\mathrm{D}_4]_{mm'} - 2\beta([\mathrm{N}]_{mm'} - [\mathrm{H}]_{mm'}) \tag{18}$$

where $\alpha \equiv \Delta t/(\mathrm{Sp}\Delta s)^4$, $\beta \equiv \mu_a\zeta/(2\mathrm{Sp}^4\Delta s^2)$, D_k are dimensionless operators corresponding

to the k-th derivative of second-order in accuracy, N is the operator containing the nonlinear terms in Eq. (2.10) and H is the operator containing the clamped conditions at the head of the flagellum. Standard centered operators are used whenever possible for D_k, but at the boundaries skewed operators are applied (*4*). The elements of N for $m = 3, \ldots, M-1$ and $m' = 1, \ldots, M+1$ take the form:

$$[\mathrm{N}]_{mm'} \quad = \quad [\mathrm{G}_2^0(n)]_{mm'} + [\mathrm{G}_0^0(n)]_{mm}[\mathrm{D}_2]_{mm'} + 2[\mathrm{G}_1^0(n)]_{mm}[\mathrm{D}_1]_{mm'} \tag{19}$$

where the operators G_k^0 are diagonal matrices with elements:

$$[\mathrm{G}_k^0(X)]_{mm'} = \delta_{mm'} D_k X_m^n \tag{20}$$

where $D_k X_m^n$ is a real number denoting the k-th derivative of the quantity X at point m at time n. The elements of H for $m = 3, \ldots, M-1$ and $m' = 1, \ldots, M+1$ read:

$$[\mathrm{H}]_{mm'} = [\mathrm{G}_2^0(n)]_{mm}[\mathrm{E}]_{mm'} \tag{21}$$

where E is a matrix with ones in the first column and zeros elsewhere. Finally, the elements b_m, $m = 3, \ldots, M-1$ read:

$$\begin{aligned} b_m \quad = \quad & \phi_m^n + \gamma[\mathrm{G}_2^0(\phi)]_{mm} - \delta[\mathrm{G}_2^0(\bar{n})]_{mm} \\ & -2\beta\{[\mathrm{G}_2^0(n)]_{mm}(\phi_m^n - \phi_1^n) + [\mathrm{G}_0^0(n)]_{mm} D_2 \phi_m^n + 2[\mathrm{G}_1^0(n)]_{mm} D_1 \phi_m^n\} \end{aligned} \tag{22}$$

where $\gamma \equiv \mu \Delta t / (\Delta s^2 \mathrm{Sp}^4)$ and $\delta \equiv \mu_a \Delta t / (\Delta s^2 \mathrm{Sp}^4)$. For $n \geq 1$, the elements of the matrix A corresponding to the rows $m = 3, \ldots, M-1$ take the form:

$$[\mathrm{A}]_{mm'} = \frac{3}{2}\delta_{mm'} + \alpha[\mathrm{D}_4]_{mm'} - 3\beta([\mathrm{N}]_{mm'} - [\mathrm{H}]_{mm'}) \tag{23}$$

The elements of N for $m = 3, \ldots, M-1$ and $m' = 1, \ldots, M+1$ read:

$$[\mathrm{N}]_{mm'} \quad = \quad [\mathrm{G}_2(n)]_{mm'} + [\mathrm{G}_0(n)]_{mm}[\mathrm{D}_2]_{mm'} + 2[\mathrm{G}_1(n)]_{mm}[\mathrm{D}_1]_{mm'} \tag{24}$$

where G_k are diagonal matrices with elements:

$$[\mathrm{G}_k(X)]_{mm'} = \delta_{mm'}(2D_k X_m^n - D_k X_m^{n-1}) \tag{25}$$

The elements of H for $m = 3, \dots, M-1$ and $m' = 1, \dots, M+1$ read:

$$[\mathrm{H}]_{mm'} = [\mathrm{G}_2(n)]_{mm}[\mathrm{E}]_{mm'} \tag{26}$$

Finally, the elements b_m, $m = 3, \dots, M-1$ read:

$$\begin{aligned} b_m &= 2\phi_m^n - \frac{1}{2}\phi_m^{n-1} + \gamma[\mathrm{G}_2(\phi)]_{mm} - \delta[\mathrm{G}_2(\bar{n})]_{mm} \\ &\quad + \beta\{[\mathrm{G}_2(n)]_{mm}[-4(\phi_m^n - \phi_1^n) + (\phi_m^{n-1} - \phi_1^{n-1})] \\ &\quad + [\mathrm{G}_0(n)]_{mm}(-4D_2\phi_m^n + D_2\phi_m^{n-1}) + 2[\mathrm{G}_1(n)]_{mm}(-4D_1\phi_m^n + D_1\phi_m^{n-1})\} \end{aligned} \tag{27}$$

The four remaining equations ($m = 1, 2, M, M+1$) are found imposing the four boundary conditions in a similar manner. The boundary conditions will be the same for $n = 0$ and $n \geq 1$ except that we will use the operators G_k^0 at $n = 0$ instead of G_k.

2.2 Dynein dynamics

Dynein dynamics is solved by using a simple implicit method for Eq. (2.11). The evolution of $n_\pm$ follows:

$$n_{\pm,m}^{n+1} = \frac{n_{\pm,m}^n + \eta\Delta t(1 - n_{\pm,m}^n)}{1 + \Delta t(1-\eta)\exp(f_{\pm,m}^n)} \tag{28}$$

where $f_{\pm,m}^n = \bar{f}[1 \mp \zeta D_t(\phi_m^n - \phi_1^n)]$ and $D_t X_m^n = (X_m^{n+1} - X_m^n)/\Delta t$.

3 Summary of variables and parameters

Table 1 shows a summary of the different parameters and variables and their corresponding symbols.

Variable	Symbol
Filament bundle centerline	$\boldsymbol{r}$
Tangent vector	$\hat{\boldsymbol{s}}$
Normal vector	$\hat{\boldsymbol{n}}$
Tangent angle	ϕ
Sliding displacement	Δ
Internal force density	f
Contact force	$\boldsymbol{N}$
Contact moment	$\boldsymbol{M}$
Plus/minus number of bound dynein motors	$n_\pm$
Load per dynein motor in the plus/minus group	$F_\pm$
Length of the flagellum	L
Axonemal diameter	b
Correlation time	τ_0
Bending stiffness	E_b
Interdoublet elastic resistance	K
Normal drag coefficient	$\zeta_\perp$
Dynein stall force	f_0
Dynein characteristic unbinding force	f_c
Dynein velocity at zero load	v_0
Dynein binding rate	π_0
Dynein unbinding rate at zero load	ε_0
Number of dynein motors in a tug-of-war unit	N
Density of tug-of-war units	ρ
Dynein activity	μ_a
Sliding resistance	μ
Sperm number	Sp
Bundle ratio	ζ
Duty ratio	η
Unbinding sensitivity ratio	$\bar{f}$

Table 1: Main variables and parameters and their corresponding symbols

References

1. S. S. Antman, *Nonlinear problems of elasticity*, vol. 107 of *Applied Math. Sci.* (Springer-Verlag New York, 1995).

2. S. Camalet, F. Jülicher, *New J. Phys.* **2**, 24 (2000).

3. U. M. Ascher, S. J. Ruuth, B. T. R. Wetton, *SIAM J. Numer. Anal.* **32**, 797 (1995).

4. A.-K. Tornberg, M. J. Shelley, *J. Comp. Phys.* **196**, 8 (2004).

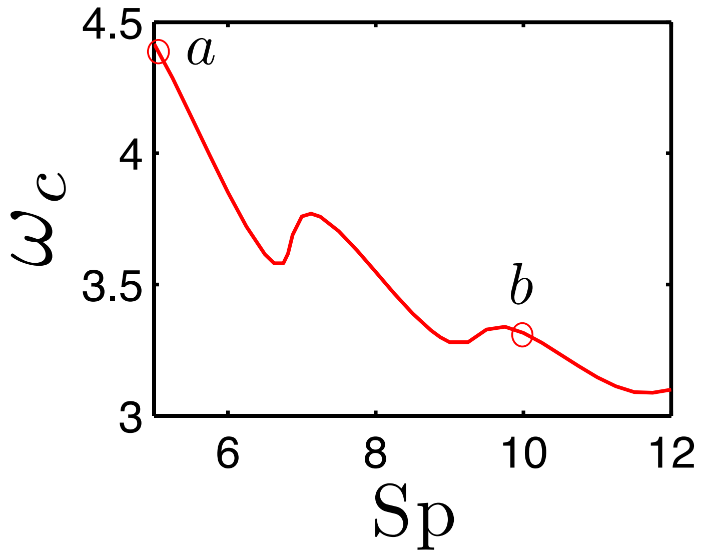

Fig. S1. Critical frequency ω_c versus Sp for the clamped condition and $\mu = 50$, $\zeta = 0.4$, $\eta = 0.14$ and $\bar{f} = 2$. The circles a and b correspond to the cases Sp $= 5$ and Sp $= 10$, respectively, from Fig. 2 in the Main Text.
